# The Obesity Drug Revolution: New Frontiers in Pharmacotherapy

**DOI:** 10.7759/cureus.96713

**Published:** 2025-11-12

**Authors:** Prabhash C Manoria

**Affiliations:** 1 Cardiology, Manoria Heart and Critical Care Hospital, Bhopal, IND

**Keywords:** glucagon-like peptide-1 receptor agonist, lifestyle modifications, metabolism, prebiotics, probiotics

## Abstract

Obesity is the most prevalent condition in high-income nations, primarily associated with increased risk of diabetes, cardiovascular disease (CVD), hypertension, and hyperlipidemia. Lifestyle modifications are a key determinant in non-pharmacological management that includes a combination of nutritional therapy, a low-calorie diet, and exercise. Earlier, anti-obesity drugs had been withdrawn from the market due to their safety profiles with cardiovascular and neuropsychiatric toxicity. The current FDA-approved pharmacotherapy consists of orlistat, setmelanotide, phentermine-topiramate, naltrexone-bupropion, liraglutide, semaglutide, and tirzepatide. Among these, semaglutide has a better clinical and regulatory profile with the feasibility of dosing and frequency. Orforglipron, a non-peptide oral glucagon-like peptide-1 receptor agonist, offers parenteral efficacy with convenient dosing. Probiotics, prebiotics, and fecal microbiota transplantation promote moderate weight loss by regulating metabolism and inflammation. Mitochondrial uncouplers help energy utilization rather than appetite regulation, which focuses on metabolic efficiency. A few challenges in obesity management are financial barriers, weight-promoting medications, inadequate obesity training, discomfort with prescribing, and lack of reimbursement. Innovative therapeutic approaches, multidisciplinary care, and a patient-centered plan are required for better clinical outcomes. This review highlights the current and emerging therapies designed to enhance long-term outcomes in obesity care.

## Introduction and background

Obesity, characterized by abnormal fat accumulation, significantly raises the risk of diabetes, cardiovascular disease, hypertension, and hyperlipidemia. It ranks as the second leading preventable cause of death after smoking and requires lifelong, multi-targeted treatment strategies [1]. Obesity arises from complex, multifactorial causes, such as genetic, environmental, psychological, nutritional, and metabolic factors, which disrupt normal adipose tissue regulation of the body [2]. As of 2021, 2.11 billion adults aged >25 years were overweight or obese, accounting for 45.1% of the global adult population, with China (402 million), India (180 million), and the USA (172 million) accounting for the majority. By 2050, projections estimate 3.8 billion affected, including 1.95 billion with obesity [3]. Prevalence is higher in high-income nations and among females [3,4]. The global economic burden is projected to reach $4.32 trillion annually by 2035 [5]. The shift from appetite suppressants to agents modulating hormonal and metabolic pathways has transformed pharmacologic management. This review summarizes current and emerging therapies designed to enhance long-term outcomes in obesity care.

## Review

Obesity management

Non-pharmacological Management

Lifestyle intervention remains the cornerstone of obesity care, encompassing diet, physical activity, and behavioral therapy, all tailored to the individual [6]. Nutritional therapy involves personalized energy-deficit diets based on clinical status and cultural factors. The American Diabetes Association (ADA) and Korean Society for the Study of Obesity (KSSO) guidelines recommend reducing daily intake by 500-750 kcal, targeting 5-10% weight loss over six months. Structured low-calorie diets (1200-1800 kcal/day) and varied macronutrient approaches, such as low-carb, low-fat, or Mediterranean diets, are effective if the caloric deficit is met. Meal replacements and culturally adapted plans are endorsed to improve adherence [7,8]. Exercise is key to weight loss and maintenance. A minimum of 150 minutes/week of moderate aerobic activity, plus two to three resistance sessions, is recommended [8]. Combined regimens outperform single modalities. Moderate-intensity workouts improve compliance, especially among sedentary individuals. Timing may influence outcomes, but requires further research [9].

Behavioral therapy enhances lifestyle change. Structured programs with ≥14 sessions in six months, including goal-setting, cognitive restructuring, and self-monitoring, are supported by the U.S. Preventive Services Task Force (USPSTF) and ADA [6,9]. Long-term follow-up beyond one year prevents weight regain, with in-person or digital formats proving effective. Adjunctive strategies like neuromodulation are under study [6]. Multidisciplinary, patient-centered plans with structured follow-up are critical for sustainable outcomes.

Pharmacological Management

Several anti-obesity drugs have been withdrawn due to post-marketing safety concerns, particularly involving cardiovascular and neuropsychiatric toxicity. Most were centrally acting agents targeting monoaminergic pathways.

Fenfluramine and dexfenfluramine, used as appetite suppressants (often combined with phentermine as “fen-phen”), were withdrawn in 1997 after being linked to valvular heart disease (VHD) and primary pulmonary hypertension (PPH). Connolly et al. reported echocardiographic abnormalities in 24% of fen-phen users versus none in controls [10]. Histology confirmed 5-HT2B-mediated valvular fibrosis [11]. The International Primary Pulmonary Hypertension Study (IPPHS) showed a 23-fold increased risk of PPH with use beyond three months [12].

Sibutramine, a serotonin-norepinephrine reuptake inhibitor, was withdrawn after SCOUT (Sibutramine Cardiovascular Outcome Trial) (n=10,744) showed a 16% increase in major cardiovascular events despite modest weight loss. This led to global withdrawal in 2010 and established cardiovascular outcome trials (CVOTs) as a regulatory requirement [13,14].

**Figure 1:**
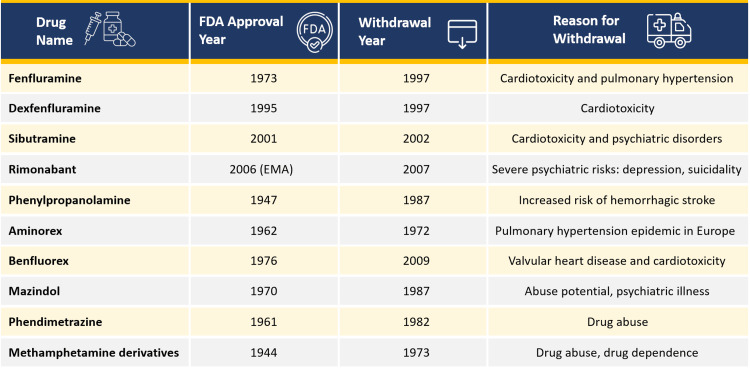
List of banned anti-obesity drugs EMA: European Medicines Agency; FDA: Food and Drug Administration. Image created by the author of this study using data obtained from [18].

Rimonabant, a CB1 receptor inverse agonist, was suspended due to psychiatric adverse events. Across the RIO (Rimonabant In Obesity and related disorders) and STRADIVARIUS (Strategy to Reduce Atherosclerosis Development Involving Administration of Rimonabant-The Intravascular Ultrasound Study) trials, rimonabant users were 2.5 times more likely to discontinue due to depression (OR: 2.5, p=0.01) and three times more likely due to anxiety (OR: 3.03, p=0.03) [15,16]. Nearly half of the adverse event-related dropouts were psychiatric, despite excluding those with prior mental illness [17]. European Medicines Agency (EMA) suspended its approval in 2008, citing risk-benefit concerns in an already vulnerable population. Other agents were withdrawn after post-marketing data confirmed unacceptable adverse effects (AEs) (Figure 1) [18].

Current pharmacotherapy

Orlistat

Orlistat, a gastrointestinal lipase inhibitor, reduces dietary fat absorption by ~30% without affecting central appetite mechanisms. It is FDA-approved for adults with a BMI ≥30 kg/m² or ≥27 kg/m² with comorbidities and is also approved for adolescents. In a two-year European trial (n=688), orlistat 120 mg TID led to 10.2% weight loss vs. 6.1% with placebo at one year and better weight maintenance in year two (2.4 kg vs. 5.0 kg regain; p<0.001), with improvements in lipids, glucose, and insulin levels [19]. A U.S. trial showed 32.8% regain vs. 58.7% in placebo (P<0.001) after diet-induced weight loss, confirming its benefit in maintenance [20]. In a 24-week Indian randomized controlled trial (RCT), orlistat reduced weight by 4.65 kg, BMI by 1.91 kg/m², and waist by 4.84 cm, with improvements in low-density lipoprotein (LDL), total cholesterol, and glucose levels [21]. In insulin-treated type 2 diabetics (n=550), orlistat showed greater weight loss (3.89 kg vs. 1.27 kg), HbA1c reduction (-0.62% vs. -0.27%), and improved glycemic and lipid profiles vs. placebo [22]. Common AEs include oily stools, fecal urgency, and GI discomfort, which usually diminish with dietary compliance. Orlistat is contraindicated in pregnancy, malabsorption, and cholestasis and may interfere with the absorption of fat-soluble vitamins and various medications. Rare cases of hepatotoxicity and oxalate nephropathy have been reported [23]. Despite modest efficacy, it remains a useful oral option for patients unsuitable for centrally acting agents or injectable therapies.

Setmelanotide

Setmelanotide, a melanocortin-4 receptor (MC4R) agonist, is the first FDA-approved treatment targeting hypothalamic leptin-melanocortin pathway defects in monogenic obesity syndromes. It is indicated for patients aged ≥2 years with obesity due to proopiomelanocortin (POMC), PCSK1, leptin receptor (LEPR) deficiencies, or Bardet-Biedl syndrome (BBS), with dosing adjusted by age and weight [24]. In the VENTURE trial (n=12; age 2-5), 83% achieved ≥0.2-point BMI Z-score reduction at week 52, with a mean BMI change of −18% (−26% in POMC/LEPR, −10% in BBS), and 91% reported reduced hunger [25]. A separate phase 3 study (NCT03746522) in BBS patients (n=38) showed ≥10% weight loss in 32.3% and improved hunger scores in 62.5%, with a mean −2.4% weight change at 14 weeks [26]. AEs include injection site reactions, hyperpigmentation (due to MC1R activation), nausea, headache, and rare psychiatric symptoms such as depression or suicidal ideation. Setmelanotide is contraindicated in patients with hypersensitivity to its components and is not approved in neonates due to benzyl alcohol toxicity. Dermatologic and psychiatric monitoring is recommended during therapy [24].

Phentermine-Topiramate

Phentermine-topiramate, a fixed-dose combination of a sympathomimetic amine and an antiepileptic, synergistically suppresses appetite and enhances satiety. FDA-approved for adults (BMI ≥30 or ≥27 kg/m² with comorbidities) and adolescents ≥12 years, its dosing is titrated up to 15 mg/92 mg daily based on response [27]. In the 56-week CONQUER trial (n=2487), mean weight loss was −8.1 kg (7.8%) and −10.2 kg (9.8%) with 7.5/46 mg and 15/92 mg doses vs. −1.4 kg placebo, with ≥10% weight loss in 48% on 15/92 mg [28]. SEQUEL (108-week extension) confirmed sustained weight loss (−10.5%) and lower type 2 diabetes mellitus (T2DM) progression [29]. In the EQUIP trial, severely obese adults lost 10.9% with 15/92 mg vs. 1.6% placebo, with cardiometabolic improvement [30]. A blood pressure (BP) monitoring study (NCT05215418) showed a 3.3 mmHg drop in 24-hr systolic BP vs. placebo and 4.7 mmHg vs. phentermine alone [31]. In adolescents (NCT03922945), BMI reductions were −10.44% (15/92 mg) and −8.11% (7.5/46 mg) with triglyceride and high-density lipoprotein cholesterol (HDL-C) improvements [32]. The EQUATE trial showed 9.2% weight loss with 15/92 mg at 28 weeks [33]. Common AEs include paresthesia, dry mouth, insomnia, and cognitive issues. It carries boxed warnings for teratogenicity and suicidality and is contraindicated in pregnancy, glaucoma, hyperthyroidism, unstable cardiac disease, and recent monoamine oxidase inhibitor (MAOI) use. Heart rate, mood, and vision should be monitored [27].

Naltrexone-Bupropion

Naltrexone-bupropion, a centrally acting fixed-dose combination, modulates the hypothalamic melanocortin system to reduce appetite and food cravings. FDA-approved for adults with BMI ≥30 or ≥27 kg/m² with comorbidities, the target dose is 32 mg/360 mg daily, titrated over four weeks [34]. In Contrave Obesity Research I (COR-I) (n=1742), NB32/360 led to −6.1% weight loss vs. −1.3% placebo, with ≥5% weight loss in 48% vs. 16%, and ≥10% in 25% vs. 6% [35]. Contrave Obesity Research II (COR-II) (n=1496) showed −6.4% vs. −1.2% weight loss, with ≥5% loss in 50.5% vs. 17.1%, and improved hunger scores [36]. COR-bupropion as an adjunct to intensive behavior modification (BMOD) (n=793) showed 9.3% weight loss with naltrexone (NB)+BMOD (behavioral modification) vs. 5.1% with placebo+BMOD, with ≥10% loss in 41.5% vs. 20.2%, confirming additive benefit [37]. A pilot study in the BED trial (Binge-Eating Disorder) (n=22) showed 45.5% of NB32/360 users achieved ≥3% weight loss vs. 0% placebo, with reduced binge episodes [38]. A meta-analysis (25 trials, n=22,165) confirmed significant weight (WMD −3.67 kg) and waist reductions (WMD −2.98 cm) vs. control, with better efficacy than bupropion alone and sustained benefit >26 weeks [39]. Common AEs include nausea, headache, insomnia, and GI symptoms; elevations in BP and heart rate (HR) necessitate monitoring. Contraindications include seizure disorder, uncontrolled hypertension, bulimia, opioid use, and recent MAOI use. The drug carries a boxed warning for suicidality, especially in young adults, and should be discontinued if serious psychiatric or hypertensive events occur [34].

Liraglutide

Liraglutide, a long-acting glucagon‑like peptide‑1 (GLP-1) receptor agonist with 97% homology to endogenous GLP-1, reduces appetite and delays gastric emptying, facilitating gradual weight loss. Approved under Saxenda for adults (BMI ≥30 or ≥27 kg/m² with comorbidities) and adolescents ≥12 years (body weight ≥60 kg), it is administered daily via subcutaneous injection, titrated to a 3.0 mg maintenance dose [40]. In the 56-week SCALE Obesity and Prediabetes trial (Effect of Liraglutide on Body Weight in Non-diabetic Obese Subjects or Overweight Subjects With Co-morbidities) (n=3731), liraglutide 3.0 mg led to 8.4 kg (8%) weight loss vs. 2.8 kg (2.6%) with placebo, with ≥5% loss in 63.2% vs. 27.1%, and ≥10% in 33.1% vs. 10.6% [41]. A pediatric trial (n=251) showed a 4.64% BMI and 4.5 kg weight reduction, with ≥5% BMI loss in 43.3% vs. 18.7% and ≥10% in 26.1% vs. 8.1% [42]. A pharmacogenomic study linked delayed gastric emptying and GLP1R/TCF7L2 variants to enhanced fat and weight loss, supporting precision therapy potential [43]. A meta-analysis (n=8249) confirmed liraglutide reduced weight (-3.35 kg), BMI (-1.45 kg/m²), HbA1c (-0.76%), and BP (SBP -3.07 mmHg, diastolic BP (DBP) -1.01 mmHg) without increased hypoglycemia [44]. Common AEs include nausea, vomiting, and GI upset; rarer effects include tachycardia, gallbladder disease, pancreatitis, and suicidal ideation. It carries a boxed warning for thyroid C-cell tumors and is contraindicated in patients with medullary thyroid carcinoma (MTC), multiple endocrine neoplasia syndrome type 2 (MEN 2), pregnancy, pancreatitis history, or severe GI disease [40]. Despite daily dosing, liraglutide remains a key option for patients needing daily therapy or unable to tolerate weekly GLP-1 analogues.

Semaglutide

Semaglutide, a long-acting GLP-1 receptor agonist approved as Wegovy, reduces appetite and caloric intake by acting on central appetite-regulating centers. It enhances glucose-dependent insulin secretion, inhibits glucagon, and delays gastric emptying, contributing to weight loss and improved glycemic control. The FDA (March 2024) approved it for weight management and for reducing major adverse cardiovascular events in adults with obesity or overweight and established cardiovascular disease (CVD). It's also indicated for pediatric obesity (age ≥12 years). Starting at 0.25 mg weekly, the dose is titrated to 2.4 mg to limit GI side effects [45].

In the STEP-1 trial (Semaglutide Treatment Effect in People with Obesity), semaglutide 2.4 mg weekly led to 14.9% weight loss over 68 weeks vs. 2.4% with placebo. Around ≥5%, ≥10%, ≥15%, and ≥20% weight loss was achieved by 86.4%, 69.1%, 50.5%, and 32%, respectively. HbA1c, waist circumference, and blood pressure also improved. At week 68, 32% of participants receiving semaglutide achieved a body-weight reduction of ≥20%, compared with only 1.7% in the placebo group (p<0.001). STEP-2 in type 2 diabetics showed a 9.6% weight loss vs. 3.4% with placebo, with significant HbA1c (−1.6%) and BP reduction [46,47].

In the STEP HFpEF trial (Semaglutide Treatment Effect in People with Obesity and Heart Failure with Preserved Ejection Fraction), 2.4 mg weekly reduced body weight by 13.3% and improved functional metrics, including Kansas City Cardiomyopathy Questionnaire - Clinical Summary Score (KCCQ-CSS) (+16.6 points), 6-minute walk distance (6MWD) (+21.5 m), and C-reactive protein (CRP) (-43.5%) vs. placebo. The diabetes subgroup in STEP HFpEF DM (STEP HFpEF Diabetes Mellitus) showed similar benefits (9.8% weight loss, +13.7 KCCQ-CSS, -42% CRP) [48,49]. The SELECT trial (Semaglutide Effects on Cardiovascular Outcomes in People with Overweight or Obesity) (n=17,604) showed a 20% reduction in cardiovascular death, myocardial infarction (MI), or stroke over 39.8 months in patients with obesity and CVD but without diabetes (6.5% vs. 8.0%, p<0.001). Semaglutide also reduced heart failure events, all-cause mortality, and revascularization, with fewer serious adverse events (33.4% vs. 36.4%) but higher discontinuation due to side effects (16.6% vs. 8.2%). The rate of hospitalization or urgent medical visits for heart failure was assessed, and semaglutide showed higher weight reduction at 104 weeks compared to placebo (-9.39% vs. -0.08%) [50].

Common adverse effects include GI symptoms (nausea, vomiting, and diarrhea), which mostly resolve over time. Serious risks involve thyroid C-cell tumors (boxed warning) [45], pancreatitis, gallbladder disease, acute kidney injury (AKI), diabetic retinopathy, increased heart rate, and mood changes, including suicidal ideation [50]. Despite these, semaglutide’s proven weight loss and cardiovascular benefits position it as a leading option in managing obesity with comorbid CVD or diabetes.

Tirzepatide

Tirzepatide is a dual GIP and GLP-1 receptor agonist that enhances insulin secretion, reduces appetite, delays gastric emptying, and improves metabolic markers. It acts on hypothalamic appetite centers and is approved (as Zepbound) for chronic weight management in adults with obesity or overweight with comorbidities. In 2024, it also received FDA approval for treating obstructive sleep apnea (OSA) in adults with obesity. Dosing starts at 2.5 mg weekly, escalating to 5, 10, or 15 mg, with 10 and 15 mg used in OSA [51].

In SURMOUNT-1 (Efficacy and Safety of Tirzepatide Once Weekly Versus Placebo in Participants Who are either Obese or Overweight with Weight-Related Comorbidities), tirzepatide led to 15.0-20.9% weight loss vs. 3.1% with placebo over 72 weeks, with >90% achieving ≥5% weight loss and >56% losing ≥20%. Waist circumference and blood pressure improved significantly. A higher dose of tirzepatide (15 mg) showed maximum waist circumference reduction (- 18.5 cm). Similarly, the change in SBP by 72 weeks was higher in the tirzepatide group (-7.2 mmHg) than in the placebo group (-1.0 mmHg). Reduction in body weight of 10%, 15%, or more was achieved mostly by a higher dose of tirzepatide (15 mg). Most of the participants on tirzepatide 15 mg showed body-weight reduction ≥20% and ≥25% at week 72, while serious AEs leading to discontinuation of the drug were mostly associated with tirzepatide 10 mg [52].

The SURMOUNT-2 showed similar results in diabetics, with 12.8-14.7% weight loss and >2% HbA1c reduction [53]. In SURMOUNT-3, with prior lifestyle intervention, 15 mg of tirzepatide led to 18.4% weight loss and major improvements in BMI and waist circumference despite non-diabetic status. The HbA1c declined by 0.5% in the tirzepatide group. A total of 87.5% of the tirzepatide group lost ≥5% of their body weight, compared to 16.5% in the placebo group, while SBP decreased by 5.1 mmHg with tirzepatide, which was increased by 4.1 mmHg in the placebo group. Serious adverse events were observed in 5.9% of the tirzepatide group and 4.8% of the placebo group. While 10.5% of participants discontinued tirzepatide due to adverse effects [54].

In the SUMMIT trial (Efficacy and Safety of Tirzepatide Versus Placebo in Patients With Heart Failure With Preserved Ejection Fraction and Obesity), 15 mg tirzepatide reduced cardiovascular death or heart failure (HF) events to 9.9% vs. 15.3% with placebo, lowered hospitalizations, improved KCCQ-CSS (+19.5 points), increased 6MWD (+26 m), and reduced CRP (-38.8%) and N-terminal pro-B-type natriuretic peptide (NT-proBNP) levels, showing potent cardiometabolic effects in HFpEF patients. The SBP was decreased by 4.6 ± 0.8 mmHg among the participants receiving tirzepatide, while in the placebo group, it increased slightly by 0.1 ± 0.8 mmHg. Around 9.9% of participants in the tirzepatide group experienced adjudicated cardiovascular death or worsening heart‑failure events, compared to 15.3% in the placebo group. Around 3.3% of participants in the tirzepatide group were hospitalized for worsening heart failure, and 1.4% of participants in the tirzepatide group required intravenous diuretic therapy in an urgent care setting. Death from any cause was reported in 5.2% of participants in the tirzepatide group and 4.1% in the placebo group [55].

Common side effects are GI-related, such as nausea, diarrhea, and vomiting, mostly during titration. Other AEs include dizziness, injection site reactions, hair loss, and fatigue. Hypoglycemia risk increases with insulin or sulfonylureas. Rare serious risks include pancreatitis, gallbladder disease, AKI, and hypersensitivity reactions. Tirzepatide carries a boxed warning for thyroid C-cell tumors and is contraindicated in patients with MTC, MEN 2, or prior serious hypersensitivity [51].

Comparison of drugs based on meta-analysis results

Meta-analysis comparisons highlight efficacy as a key differentiator among anti-obesity drugs. Orlistat offers the lowest mean weight loss (~10.2%) with minimal impact beyond lipid levels, while naltrexone-bupropion (~6.1%) and liraglutide (~8%) show modest efficacy. Phentermine-topiramate achieves ~10.9%, semaglutide delivers superior efficacy (14.9%), and tirzepatide leads at 20.9%, though it lacks FDA approval for CV risk reduction or adolescent use [56]. Semaglutide shows robust efficacy across populations, with >85% achieving ≥5% weight loss and significant improvements in HbA1c (-1.6%), SBP (-6.2 mmHg), and waist circumference (-13.5 cm). Tirzepatide shows similar gains (HbA1c -1.69%) but without a CV indication. Liraglutide provides moderate benefits (HbA1c -1.09%, SBP -2.4 mmHg). Adherence is lowest with orlistat (0%) and naltrexone-bupropion (10%), moderate with phentermine-topiramate (13%) and liraglutide (17%), and highest with semaglutide (40%), aided by weekly dosing and tolerability. Semaglutide is also the only agent FDA-approved for CV risk reduction and adolescent obesity, with the added advantage of oral formulation. Tirzepatide's adherence appears promising but remains under evaluation. Regulatory limitations apply to phentermine-topiramate (teratogenicity) and naltrexone-bupropion (psychiatric risk). Overall, semaglutide offers the most favorable clinical and regulatory profile, combining strong efficacy, cardiometabolic benefits, weekly and oral options, and broad approval across risk groups.

Pipeline drugs

The anti-obesity drug pipeline (Table 1) is rapidly expanding with next-generation agents targeting pathways beyond traditional mono-hormonal strategies. Oral GLP-1 receptor agonists like orforglipron are advancing into late-phase trials. In ACHIEVE-1, a 40-week, phase 3 study in adults with type 2 diabetes, Orforglipron (3-36 mg) reduced HbA1c by 1.3-1.6% versus 0.1% with placebo, with >65% of participants on the highest dose achieving HbA1c ≤6.5%. Mean weight loss at the highest dose reached 7.3 kg (7.9%), without plateauing by week 40.

**Table 1:** Anti-obesity drugs in the development pipeline Amylin RA: amylin receptor agonist; CKD: chronic kidney disease; CV: cardiovascular; GCG RA: glucagon receptor agonist; GIP RA: glucose‑dependent insulinotropic peptide receptor agonist; GLP‑1 RA: glucagon‑like peptide‑1 receptor agonist; GLP‑2 RA: glucagon‑like peptide‑2 receptor agonist; MASLD: metabolic dysfunction‑associated steatotic liver disease; MASH: metabolic dysfunction‑associated steatohepatitis; OA: osteoarthritis; T2D/T2DM: type 2 diabetes mellitus Table created by the author of this study using data obtained from [57].

S. Np	Drug/Molecule	Company	Mech. Of Action	Indications Other Than Obesity	Obesity Trial
1	Orforglipron	Eli Lilly	GLP-1 RA	T2D, CV outcomes in T2D (Phase-3)	Phase-3
2	CagriSema (Cagrilintide + Semaglutide	Novo Nordisk	GLP-1 RA + Amylin RA	T2D, CV outcomes in T2D (Phase-3)	Phase-3
3	Survodutide	Boehringer Ingelheim	GLP-1 RA + GCG RA	T2DM, Obesity (Phase-2)	Phase-3
5	Retatrutide	Eli Lilly	GLP-1 RA + GIP RA + GCG RA	T2D, OA (Phase-3), CKD (Phase-1)	Phase-3
6	Danuglipron	Pfizer	GLP-1 RA	Obesity, T2DM	Phase-2
7	Efinopegdutide	Hanmi Pharma	GLP-1 RA + GCG RA	T2D, MASH, MASLD (Phase-2)	Phase-2
8	Maridebart Cafraglutide (AMG 133)	Amgen	GLP-1 RA + GIPR Antagonist	Obesity, T2DM	Phase-2
9	Dapiglutide	Zealand Pharma	GLP-1 RA + GLP 2 RA	Obesity	Phase-2
10	ZP8396	Zealand Pharma	Amylin RA	Obesity	Phase-1
11	VK2735	Viking Therapeutics	GLP-1 RA + GIP RA	MASH (Phase-1)	Phase-1
12	Amycretin	Novo Nordisk	GLP-1 RA + Amylin RA	Obesity	Phase-1
13	HM15211	Hanmi Pharma	GLP-1 RA + GIP RA + GCG RA	(Obesity) MASH (Phase-2)	Phase-1
14	Petrelintide	Zealand Pharma	Amylin Analogue	Obesity	Phase-1
15	Eloralintide	Eli Lilly	Amylin Analogue	Obesity	Phase-2

GI-related adverse events were the most common (e.g., diarrhea 26%, nausea 18%), but discontinuation rates remained low (4-8%) [58]. These results establish orforglipron as a promising oral, non-peptide GLP-1 option, combining injectable-level efficacy with convenient dosing. Other high-potential agents include retatrutide (a triple GLP-1/GIP/glucagon agonist), CagriSema (semaglutide + amylin analog), and bimagrumab + semaglutide, which uniquely preserves lean mass via ActRII inhibition. Agents like mazdutide, survodutide, and pemvidutide pair GLP-1 with glucagon receptor activity, targeting both obesity and metabolic dysfunction‑associated steatohepatitis (MASH). Amycretin, a dual GLP-1/amylin oral agent, and emerging non-incretin candidates like GDF15 analogues, PYY agonists, and mitochondrial-targeted compounds broaden the therapeutic scope. Companies including Novo Nordisk, Eli Lilly, and Amgen are spearheading development, with over 30 agents across various stages [59]. Collectively, these innovations could rival bariatric outcomes and transform obesity care through scalable, multi-pathway pharmacotherapy.

Role of gut microbiome in obesity management

The gut microbiome influences obesity by regulating metabolism, appetite, and inflammation. Dysbiosis, particularly an altered firmicutes-to-bacteroidetes ratio, enhances energy extraction and fat accumulation. Microbial metabolites like short-chain fatty acids (SCFAs) improve insulin sensitivity and gut barrier function, while dysbiosis can promote systemic inflammation via lipopolysaccharides (LPS) and cytokines like IL-6 and TNF-α [60,61]. Animal studies show microbiota from obese donors induces weight gain, and obese individuals often have reduced microbial diversity and fewer beneficial bacteria. Interventions like probiotics, prebiotics, and fecal microbiota transplantation are under investigation, showing promise for modest weight loss and metabolic benefits as adjuncts in obesity care [60,61].

Role of mitochondrial uncouplers in obesity treatment

Mitochondrial uncouplers offer a novel obesity treatment strategy by increasing energy expenditure rather than suppressing appetite. They disrupt the mitochondrial proton gradient, boosting oxygen consumption and fat oxidation. BAM15, a next-generation uncoupler, reduced fat mass, improved insulin sensitivity, and lowered liver fat in obese mice without affecting food intake or causing side effects [62]. SHD865, a derivative with better pharmacokinetics, further improved glucose tolerance and suppressed lipogenesis by downregulating acetyl-CoA carboxylase (ACC) and fatty acid synthase (FAS) enzymes [63]. These agents target metabolic efficiency directly, showing promise as safe, effective therapies to complement or replace current appetite-focused drugs in obesity management.

Challenges in obesity management

Despite growing therapeutic advances, obesity management remains hampered by challenges from both patients and healthcare systems. Many people with obesity (PwO) still view it as a personal failure rather than a chronic disease, leading to poor long-term adherence, with fewer than 10% persisting with pharmacotherapy beyond a year. Misinformation from social circles and social media, cultural pressures, financial barriers, comorbidities, and weight-promoting medications further complicate efforts. On the clinician side, limited consultation time, inadequate obesity training, discomfort with prescribing, lack of reimbursement, and systemic weight bias hinder care delivery. Structural misalignment and policy gaps also reduce access to consistent, evidence-based obesity treatment [64].

## Conclusions

Obesity is a chronic, relapsing disease associated with significant metabolic and cardiovascular consequences. Recent pharmacologic advancements, particularly with incretin-based therapies such as tirzepatide, a dual GIP and GLP-1 receptor agonist, have redefined the landscape of obesity management. Both tirzepatide and semaglutide, GLP-1 receptor agonists, have demonstrated excellent efficacy and safety in the management of both T2DM and obesity. With these agents now available for clinical use, they set a new benchmark in pharmacologic obesity treatment. As the global burden of obesity continues to rise, ensuring access to effective therapies like semaglutide and tirzepatide will be crucial. Addressing the multifaceted nature of obesity will require continued therapeutic innovation, a multidisciplinary care model, and a patient-centered approach to achieve sustained, meaningful outcomes.

## References

[1] Panuganti KK, Nguyen M, Kshirsagar RK (2025). Obesity (Nursing). StatPearls [Internet].

[2] Rubino F, Cummings DE, Eckel RH (2025). Definition and diagnostic criteria of clinical obesity. Lancet Diabetes Endocrinol.

[3] (2025). Global, regional, and national prevalence of adult overweight and obesity, 1990-2021, with forecasts to 2050: a forecasting study for the Global Burden of Disease Study 2021. Lancet.

[4] Islam AN, Sultana H, Nazmul Hassan Refat M, Farhana Z, Abdulbasah Kamil A, Meshbahur Rahman M (2024). The global burden of overweight-obesity and its association with economic status, benefiting from STEPs survey of WHO member states: a meta-analysis. Prev Med Rep.

[5] Okunogbe A, Nugent R, Spencer G, Powis J, Ralston J, Wilding J (2022). Economic impacts of overweight and obesity: current and future estimates for 161 countries. BMJ Glob Health.

[6] Higuera-Hernández MF, Reyes-Cuapio E, Gutiérrez-Mendoza M (2018). Fighting obesity: non-pharmacological interventions. Clin Nutr ESPEN.

[7] ElSayed NA, Aleppo G, Bannuru RR (2024). Pharmacologic approaches to glycemic treatment: standards of care in diabetes-2024. Diabetes Care.

[8] Kim KK, Haam JH, Kim BT (2023). Evaluation and treatment of obesity and its comorbidities: 2022 update of clinical practice guidelines for obesity by the Korean Society for the study of obesity. J Obes Metab Syndr.

[9] Wadden TA, Tronieri JS, Butryn ML (2020). Lifestyle modification approaches for the treatment of obesity in adults. Am Psychol.

[10] Connolly HM, Crary JL, McGoon MD, Hensrud DD, Edwards BS, Edwards WD, Schaff HV (1997). Valvular heart disease associated with fenfluramine-phentermine. N Engl J Med.

[11] Rothman RB, Baumann MH (2009). Serotonergic drugs and valvular heart disease. Expert Opin Drug Saf.

[12] Abenhaim L, Moride Y, Brenot F (1996). Appetite-suppressant drugs and the risk of primary pulmonary hypertension. International Primary Pulmonary Hypertension Study Group. N Engl J Med.

[13] James WP, Caterson ID, Coutinho W (2010). Effect of sibutramine on cardiovascular outcomes in overweight and obese subjects. N Engl J Med.

[14] James WT (2005). The SCOUT study: risk-benefit profile of sibutramine in overweight high-risk cardiovascular patients. EHJ-S.

[15] Van Gaal LF, Scheen AJ, Rissanen AM, Rössner S, Hanotin C, Ziegler O (2008). Long-term effect of CB1 blockade with rimonabant on cardiometabolic risk factors: two year results from the RIO-Europe Study. Eur Heart J.

[16] Nissen SE, Nicholls SJ, Wolski K (2008). Effect of rimonabant on progression of atherosclerosis in patients with abdominal obesity and coronary artery disease: the STRADIVARIUS randomized controlled trial. JAMA.

[17] Christensen R, Kristensen PK, Bartels EM, Bliddal H, Astrup A (2007). Efficacy and safety of the weight-loss drug rimonabant: a meta-analysis of randomised trials. Lancet.

[18] Onakpoya IJ, Heneghan CJ, Aronson JK (2016). Post-marketing withdrawal of anti-obesity medicinal products because of adverse drug reactions: a systematic review. BMC Med.

[19] Sjöström L, Rissanen A, Andersen T (1998). Randomised placebo-controlled trial of orlistat for weight loss and prevention of weight regain in obese patients. Lancet.

[20] Hill JO, Hauptman J, Anderson JW (1999). Orlistat, a lipase inhibitor, for weight maintenance after conventional dieting: a 1-y study. Am J Clin Nutr.

[21] Jain SS, Ramanand SJ, Ramanand JB, Akat PB, Patwardhan MH, Joshi SR (2011). Evaluation of efficacy and safety of orlistat in obese patients. Indian J Endocrinol Metab.

[22] Kelley DE, Bray GA, Pi-Sunyer FX, Klein S, Hill J, Miles J, Hollander P (2002). Clinical efficacy of orlistat therapy in overweight and obese patients with insulin-treated type 2 diabetes: a 1-year randomized controlled trial. Diabetes Care.

[23] (2025). Orlistat FDA Drug Label. https://www.accessdata.fda.gov/drugsatfda_docs/label/2009/020766s026lbl.pdf.

[24] (2025). Setmelanotide FDA Drug Label. https://www.accessdata.fda.gov/drugsatfda_docs/label/2022/213793s001lbl.pdf.

[25] Argente J, Verge CF, Okorie U (2025). Setmelanotide in patients aged 2-5 years with rare MC4R pathway-associated obesity (VENTURE): a 1 year, open-label, multicenter, phase 3 trial. Lancet Diabetes Endocrinol.

[26] Haqq AM, Chung WK, Dollfus H (2022). Efficacy and safety of setmelanotide, a melanocortin-4 receptor agonist, in patients with Bardet-Biedl syndrome and Alström syndrome: a multicentre, randomised, double-blind, placebo-controlled, phase 3 trial with an open-label period. Lancet Diabetes Endocrinol.

[27] (2025). Phentermine and Topiramate FDC FDA Drug Label. https://www.accessdata.fda.gov/drugsatfda_docs/label/2024/022580s025lbl.pdf.

[28] Gadde KM, Allison DB, Ryan DH (2011). Effects of low-dose, controlled-release, phentermine plus topiramate combination on weight and associated comorbidities in overweight and obese adults (CONQUER): a randomised, placebo-controlled, phase 3 trial. Lancet.

[29] Garvey WT, Ryan DH, Look M (2012). Two-year sustained weight loss and metabolic benefits with controlled-release phentermine/topiramate in obese and overweight adults (SEQUEL): a randomized, placebo-controlled, phase 3 extension study. Am J Clin Nutr.

[30] Allison DB, Gadde KM, Garvey WT (2012). Controlled-release phentermine/topiramate in severely obese adults: a randomized controlled trial (EQUIP). Obesity (Silver Spring).

[31] Bays HE, Hsia DS, Nguyen LT, Peterson CA, Varghese ST (2024). Effects of phentermine / topiramate extended-release, phentermine, and placebo on ambulatory blood pressure monitoring in adults with overweight or obesity: a randomized, multicenter, double-blind study. Obes Pillars.

[32] Kelly AS, Bensignor MO, Hsia DS, Shoemaker AH, Shih W, Peterson C, Varghese ST (2022). Phentermine/topiramate for the treatment of adolescent obesity. NEJM Evid.

[33] Aronne LJ, Wadden TA, Peterson C, Winslow D, Odeh S, Gadde KM (2013). Evaluation of phentermine and topiramate versus phentermine/topiramate extended-release in obese adults. Obesity (Silver Spring).

[34] (2025). Naltrexone and Bupropion FDC Combination FDA Drug Label. https://www.accessdata.fda.gov/drugsatfda_docs/label/2021/200063s020lbl.pdf.

[35] Greenway FL, Fujioka K, Plodkowski RA (2010). Effect of naltrexone plus bupropion on weight loss in overweight and obese adults (COR-I): a multicentre, randomised, double-blind, placebo-controlled, phase 3 trial. Lancet.

[36] Apovian CM, Aronne L, Rubino D (2013). A randomized, phase 3 trial of naltrexone SR/bupropion SR on weight and obesity-related risk factors (COR-II). Obesity (Silver Spring).

[37] Wadden TA, Foreyt JP, Foster GD (2011). Weight loss with naltrexone SR/bupropion SR combination therapy as an adjunct to behavior modification: the COR-BMOD trial. Obesity (Silver Spring).

[38] Grilo CM, Lydecker JA, Morgan PT, Gueorguieva R (2021). Naltrexone + bupropion combination for the treatment of binge-eating disorder with obesity: a randomized, controlled pilot study. Clin Ther.

[39] Liu Y, Han F, Xia Z, Sun P, Rohani P, Amirthalingam P, Sohouli MH (2024). The effects of bupropion alone and combined with naltrexone on weight loss: a systematic review and meta-regression analysis of randomized controlled trials. Diabetol Metab Syndr.

[40] (2025). Liraglutide FDA Drug Label. https://www.accessdata.fda.gov/drugsatfda_docs/label/2017/022341s027lbl.pdf.

[41] Pi-Sunyer X, Astrup A, Fujioka K (2015). A randomized, controlled trial of 3.0 mg of liraglutide in weight management. N Engl J Med.

[42] Kelly AS, Auerbach P, Barrientos-Perez M (2020). A randomized, controlled trial of liraglutide for adolescents with obesity. N Engl J Med.

[43] Maselli D, Atieh J, Clark MM (2022). Effects of liraglutide on gastrointestinal functions and weight in obesity: a randomized clinical and pharmacogenomic trial. Obesity (Silver Spring).

[44] Barboza JJ, Huamán MR, Melgar B, Diaz-Arocutipa C, Valenzuela-Rodriguez G, Hernandez AV (2022). Efficacy of liraglutide in non-diabetic obese adults: a systematic review and meta-analysis of randomized controlled trials. J Clin Med.

[45] (2025). Semaglutide FDA Drug Label. https://www.accessdata.fda.gov/drugsatfda_docs/label/2023/215256s007lbl.pdf.

[46] Wilding JP, Batterham RL, Calanna S (2021). Once-weekly semaglutide in adults with overweight or obesity. N Engl J Med.

[47] Davies M, Faerch L, Jeppesen OK (2021). Semaglutide 2.4 mg once a week in adults with overweight or obesity, and type 2 diabetes (STEP 2): a randomised, double-blind, double-dummy, placebo-controlled, phase 3 trial. Lancet.

[48] Kosiborod MN, Abildstrøm SZ, Borlaug BA (2023). Semaglutide in patients with heart failure with preserved ejection fraction and obesity. N Engl J Med.

[49] Kosiborod MN, Petrie MC, Borlaug BA (2024). Semaglutide in patients with obesity-related heart failure and type 2 diabetes. N Engl J Med.

[50] Lincoff AM, Brown-Frandsen K, Colhoun HM (2023). Semaglutide and cardiovascular outcomes in obesity without diabetes. N Engl J Med.

[51] (2025). Tirzepatide FDA Drug Label. https://www.accessdata.fda.gov/drugsatfda_docs/label/2024/217806s013lbl.pdf.

[52] Jastreboff AM, Aronne LJ, Ahmad NN (2022). Tirzepatide once weekly for the treatment of obesity. N Engl J Med.

[53] Garvey WT, Frias JP, Jastreboff AM (2023). Tirzepatide once weekly for the treatment of obesity in people with type 2 diabetes (SURMOUNT- 2): a double-blind, randomised, multicentre, placebo-controlled, phase 3 trial. Lancet.

[54] Wadden TA, Chao AM, Machineni S (2023). Tirzepatide after intensive lifestyle intervention in adults with overweight or obesity: the SURMOUNT-3 phase 3 trial. Nat Med.

[55] Packer M, Zile MR, Kramer CM (2025). Tirzepatide for heart failure with preserved ejection fraction and obesity. N Engl J Med.

[56] Gudzune KA, Kushner RF (2024). Medications for obesity: a review. JAMA.

[57] Grandl G, Novikoff A, Liu X, Müller TD (2024). Recent achievements and future directions of anti-obesity medications. Lancet Reg Health Eur.

[58] (2025). Lilly’s Oral GLP-1, Orforglipron, Demonstrated Statistically Significant Efficacy Results and a Safety Profile Consistent With Injectable GLP-1 Medicines in Successful Phase 3 Trial. https://investor.lilly.com/news-releases/news-release-details/lillys-oral-glp-1-orforglipron-demonstrated-statistically.

[59] Melson E, Ashraf U, Papamargaritis D, Davies MJ (2025). What is the pipeline for future medications for obesity?. Int J Obes.

[60] Yarahmadi A, Afkhami H, Javadi A, Kashfi M (2024). Understanding the complex function of gut microbiota: its impact on the pathogenesis of obesity and beyond: a comprehensive review. Diabetol Metab Syndr.

[61] Aoun A, Darwish F, Hamod N (2020). The influence of the gut microbiome on obesity in adults and the role of probiotics, prebiotics, and synbiotics for weight loss. Prev Nutr Food Sci.

[62] Alexopoulos SJ, Chen SY, Brandon AE (2020). Mitochondrial uncoupler BAM15 reverses diet-induced obesity and insulin resistance in mice. Nat Commun.

[63] Ramshankar G, Perry RJ (2024). A new mitochondrial uncoupler improves metabolic homeostasis in mice. Diabetes.

[64] Kim TN (2020). Barriers to obesity management: patient and physician factors. J Obes Metab Syndr.

